# Anti‐*Helicobacter pylori* activity of *Fagopyrum Tataricum* (L.) Gaertn. Bran flavonoids extract and its effect on *Helicobacter pylori*‐induced inflammatory response

**DOI:** 10.1002/fsn3.3329

**Published:** 2023-03-31

**Authors:** Liting Qing, Shu Li, Shiying Yan, Chengmeng Wu, Xin Yan, Zongyu He, Qian Chen, Min Huang, Caihong Shen, Songtao Wang, Mei Cao, Jian Zhao

**Affiliations:** ^1^ Key Laboratory of Biological Resource and Ecological Environment of Chinese Education Ministry, College of Life Sciences Sichuan University 610064 Chengdu China; ^2^ Luzhou Pinchuang Technology Co., Ltd. (National Engineering Research Center of Solid‐state Brewing) 646000 Luzhou China; ^3^ Core Laboratory, School of Medicine Sichuan Provincial People's Hospital Affiliated to University of Electronic Science and Technology of China 610072 Chengdu China; ^4^ Key Laboratory of Irradiation Preservation of Sichuan Province Sichuan Institute of Atomic Energy Sichuan Chengdu China

**Keywords:** *Fagopyrum Tataricum* bran, *Helicobacter pylori*, inflammation, Total flavonoids

## Abstract

Tartary buckwheat flavonoids have a variety of effects on anti‐inflammatory, anti‐oxidation, as well as anti‐tumor and are valuable for academic research and industrial application. *Helicobacter pylori* (*H*. *pylori*) infection is associated with various gastrointestinal diseases in humans, and an increase in its resistance has led to the failure of many drugs. In this study, we quantified the main monomers of tartary buckwheat (*Fagopyrum Tataricum* (L.) Gaertn.) bran flavonoids extract through HPLC analysis. Then, we investigated the anti‐*H*. *pylori* activity and the effect on cell inflammation of tartary buckwheat flavonoids extract and its four main flavonoid monomers (rutin, quercetin, kaempferol, and nicotiflorin). The results showed that tartary buckwheat flavonoids extract and its four flavonoid monomers could inhibit the growth of *H*. *pylori* and down‐regulate the expression of proinflammatory factors IL‐6, IL‐8, and CXCL‐1 in *H*. *pylori*‐induced GES‐1 cells. Moreover, we also confirmed that tartary buckwheat flavonoids extract could reduce the expression of virulence factor gene of *H*. *pylori*. In summary, tartary buckwheat can alleviate the cell inflammation induced by *H*. *pylori*, which provides a theoretical basis for the development of tartary buckwheat healthcare products.

## Graphical abstract

This study found that tartary buckwheat (*Fagopyrum tataricum* (L.) Gaertn.) flavonoids extract and its main monomers could not only inhibit the growth of *H*. *pylori* in vitro, but also reduce the inflammatory response induced by *H*. *pylori* in GES‐1 cells. Moreover, the flavonoids extract could inhibit the expression of urease. The results provided a new idea for the development of tartary buckwheat flavonoids as health functional products.

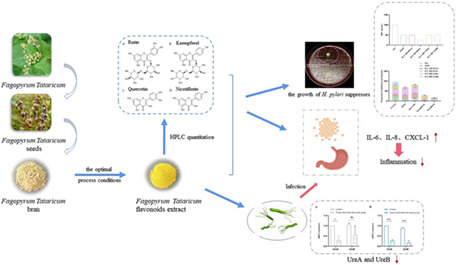

## 1 INTRODUCTION

*Helicobacter pylori* (*H*. *pylori*) is gram‐negative bacteria that planted in the stomach and duodenum of humans and may cause gastritis, peptic ulcers, and gastric cancer (Kim et al., 2021). It is prevalent in about 50% of the global population and is a major carcinogen of gastric cancer (Huang et al., 2021). Proinflammatory cytokines such as IL‐6, IL‐8, and CXCL‐1 in gastric epithelial cells and immune cells can be promoted by *H*. *pylori i*nfection (Wu et al., 2017). Various virulence factors produced by *H*. *pylori* induce the production of these cytokines (Alzahrani et al., 2014). Reducing or eradicating the inflammatory reaction will help promote ulcer healing and reduce the risk of gastric cancer. Today, first‐line standard triple therapy of two antibiotics and a proton‐pump inhibitor is the most widely used treatment to eradicate *H*. *pylori* infection. However, this approach is costly, has significant side effects (such as nausea, vomiting, and diarrhea), and increases *H*. *pylori* resistance because of the overuse of antibiotics (Boyanova et al., 2016; Fallone et al., 2016; González et al., 2021). Therefore, seeking alternative therapies to reduce *H*. *pylori* infection and treat gastrointestinal diseases is the direction that researchers continue to explore.

As early as 1982, doctors had been using natural products based on empirical knowledge to combat these diseases (Yu et al., 2022). Research suggests that over 240 plant species have shown anti‐*H*. *pylori* activity (Salehi et al., 2018). Tartary buckwheat (*Fagopyrum Tataricum* (L.) Gaertn.) is a food crop with rich nutritional value and widely distributed throughout southwest China (Tomotake et al., 2006). Its planting area and production in China is the highest in the world, and it has been popularized in many countries around the world (Holasova et al., 2002). As raw material for food, *Fagopyrum Tataricum* has been processed into buckwheat tea, flour, and noodles (Zhang et al., 2012). There are many bioactive components in tartary buckwheat, among which flavonoids are the most important ones. Flavonoids, rich in fruits, vegetables, tea, and medicinal plants, are highly effective antioxidants and have received great attention and extensive research (Rodríguez De Luna et al., 2020). Flavonoids also have the functions of preventing cardiovascular and cerebrovascular diseases, resisting cancer, anti‐aging, analgesic, and hemostasis (Chen & Zhang, 2021). In addition, studies have reported that some flavonoids (especially chalcones) show up to sixfold stronger antibacterial activities than standard drugs in the market (Farhadi et al., 2019). Kaempferol exerts a positive influence on the inflammatory response caused by *H*. *pylori* infection and could down‐regulate proinflammatory cytokines (Yeon et al., 2019). However, no studies have focused on the antibacterial activity and anti‐inflammatory properties of *Fagopyrum Tataricum* bran extract against *H*. *pylori*.

In this article, we prepared *Fagopyrum Tataricum* bran flavonoids extract under optimal extraction conditions and quantified the main flavonoid monomers in it. Then, we investigated their antibacterial activity and anti‐inflammatory properties against *H*. *pylori* in human gastric epithelial cells (GES‐1 cells).

## 2 MATERIALS AND METHODS

### 2.1 Extraction and identification of flavonoids

In preliminary experiments, we determined the optimal macroporous resin ADS‐7 and the optimal separation and purification process of tartary buckwheat bran flavonoids (the sample flow rate was 2 BV/h, the concentration of the sample solution was 1.0 mg/mL, the washing volume was 2 BV, the eluent concentration of food grade ethanol was 90%, the elution flow rate was 2 BV/h, and the elution volume was 6 BV). We validated the process by magnifying it 24 times (stage two) and 240 times (stage three), respectively. Besides, we detected standard solutions of the four flavonoid monomers by the UV–Visible Spectrum ranging from 200 nm to 500 nm, respectively, and selected the appropriate wavelength according to the maximum absorption peak. A high‐performance liquid chromatography (HPLC) system (LC‐16, Shimadzu) was used to quantitative determination of main components in tartary buckwheat bran flavonoids extract.

Regarding HPLC detection, 20 μL extract was analyzed with an Intersustain C18 column (4.6 mm × 250 mm, 5 μm). The conditions were as follows: Solvent A (acetonitrile) and solvent B (water in 0.2% phosphoric acid) were prepared. Gradient elution used was 0–25 min, 15%–25% A; 25–35 min, 25%–50% A; 35–45 min, 50%–90% A; 45–55 min, 90% A; 55–56 min, 90%–15% A; 56–60 min, 15% A. 1.0 mL/min and 35°C were used for the flow rate of mobile phase and column temperature, respectively. Mixed standards of rutin, quercetin, kaempferol, and nicotiflorin and all sample solutions were carried out at the appropriate wavelength with the HPLC system.

### 2.2 *H*. *pylori* strains and culture conditions

The two strains of *H*. *pylori* (SS1 and 26695) were derived from Key Laboratory of Biological Resource and Ecological Environment of Chinese Education Ministry. Four clinical *H*. *pylori* strains (SCU‐HP‐0916A, SCU‐HP‐0916C, SCU‐HP‐1230A, and SCU‐HP‐1230B) were from Sichuan Provincial People's Hospital. All *H*. *pylori* strains were cultured on an anaerobic incubator (5% O_2_, 10% CO_2_, and 85% N_2_) at 37°C for 3 days with a Columbia blood agar plate containing brain heart infusion broth, 7% defibrinated sheep blood, and 0.1% antibiotics (Wu et al., 2020).

### 2.3 Determination of inhibition zones

Inhibitory zones were determined by the disk diffusion antimicrobial susceptibility test. Dimethyl sulfoxide (DMSO) was used as a solvent to prepare the vehicle and as the blank control group. The concentrations of tartary buckwheat flavonoids extract and the four flavonoid monomers (rutin, quercetin, kaempferol, and nicotiflorin) in paper disks were 200 mg/mL and 50 mg/mL, respectively. After a 3‐day cultivation, the *H*. *pylori* of the blood agar plate was eluted into 1 mL sterile saline. Bacterium solution was diluted to a turbidity of 1 MacFarlane (∼3 × 10^8^ CFU/mL) by nephelometer (WGZ‐2XJ). Next, a 90 μL of the diluted suspension was swabbed evenly over the surface of a new blood agar plate with a sterile cotton swab. After slightly drying, put the paper disks to the surface of the plates and then culture at 37°C for 3 days. The inhibitory zone diameter was measured by criss‐cross method and each set of experiments was performed in triplicate.

### 2.4 Determination of minimum inhibitory concentration (MIC)

The MIC of tartary buckwheat flavonoids extract against *H*. *pylori* strains was determined by the agar dilution method. Flavonoids extracts from tartary buckwheat were added to a 24‐well plate containing 2 mL Columbia medium at the concentrations of 200, 100, 50, 25, 12.5, and 6.25 mg/mL, respectively. Blank control group and negative control group were blood plates without tartary buckwheat flavonoids extract. After the plates were solidified, 100 μL of bacterial suspension (1 × 10^8^ CFU/mL) was added to each well of the experimental group and the negative control group. Finally, the plates were incubated at 37°C in an anaerobic incubator for 3 days, and the lowest concentration at which showed *H*. *pylori* growth was considered as MIC. Each set of experiments was performed in triplicate.

### 2.5 Cell culture

The source of human gastric epithelial cells (GES‐1 cells) and their culture conditions refer to the article by Li et al. (2020).

### 2.6 Treatment of GES‐1 cells with *H*. *pylori*, tartary buckwheat flavonoids extract, and flavonoid monomers

3 × 10^5^ cells were seeded in 12‐well plates in each well. The experiment was divided into six groups in triplicate: blank control group, *H*. *pylori* group, tartary buckwheat flavonoids group, flavonoids treatment group, flavonoids prevention group, and flavonoids and *H*. *pylori* co‐treatment group. The blank control group: containing only GES‐1 cells suspension. The *H*. *pylori* group: GES‐1 cells were infected with *H*. *pylori* at a multiplicity of infection (MOI) of 100:1. The tartary buckwheat flavonoids group: The concentrations of tartary buckwheat flavonoids extract and four flavonoid monomers in GES‐1 cell suspension were 500 μg/mL and 100 μg/mL, respectively. The flavonoids treatment group: After *H*. *pylori* infecting cells at a MOI of 100:1 for 2 h, fresh medium was changed, then added 500 μg/mL tartary buckwheat flavonoids extract and 100 μg/mL four flavonoid monomers. The flavonoids prevention group: After adding 500 μg/mL tartary buckwheat flavonoids extract and 100 μg/mL four flavonoid monomers for 2 h, fresh medium was changed, then *H*. *pylori* was added to cells at a MOI of 100:1. The flavonoids and *H*. *pylori* co‐treatment group: 500 μg/mL tartary buckwheat flavonoids extract and 100 μg/mL four flavonoid monomers were added in GES‐1 cells suspension; at the same time, *H*. *pylori* was infected with the MOI of 100:1. All groups were cultured at 37°C with 5% CO_2_.

### 2.7 Quantitative real‐time PCR (qRT‐PCR)

Total RNAs of *H*. *pylori* and cells were extracted with Trizol reagent. RNA concentration was determined using BioDrop, and then cDNA was obtained by reverse transcription using PrimeScript RT reagent Kit (RR047A, TaKaRa). qRT‐PCR was performed using an iQ5 Real‐Time PCR detection system (Quant Studio 3, Thermo). The amplification was programed to start at 95°C for 30 s, 40 cycles of degeneration at 95°C for 15 s, annealing at 60°C for 30 s, and extension at 72°C for 20 s. The primers were designed using Primer 5.0 software and are listed in Table 1.

**TABLE 1:** qRT‐PCR primer sequences.

Name	Sequence (5′–3′)
gyrB (*H*. *pylori*)	
Sense:	CCCTAACGAAGCCAAAATCA
Antisense:	GCACTATCGCCCTCCACTAA
UreA (*H*. *pylori*)	
Sense:	TCAAACCTTACCGCTGTCCC
Antisense:	CGGTTCAAATCGGCTCACAC
UreB (*H*. *pylori*)	
Sense:	GCCAGCGATTTTGCCATCTT
Antisense:	GTGGCGGTAAAACCCTGAGA
IL‐6 (hsa)	
Sense	CTTCGGTCCAGTTGCCTTCT
Antisense	TGGAATCTTCTCCTGGGGGT
IL‐8 (hsa)	
Sense	ACACTGCGCCAACACAGAAA
Antisense	CAACCCTCTGCACCCAGTTT
CXCL‐1 (hsa)	
Sense	ATTTCTGAGGAGCCTGCAAC
Antisense	CACATACATTCCCCTGCCTT
GAPDH (hsa)	
Sense	TGCACCACCAACTGCTTAGC
Antisense	GGCATGGACTGTGGTCATGAG

### 2.8 Statistical analysis

All analysis results were described as mean ± standard deviation (SD). The data were analyzed with GraphPad Prism 8.0 (GraphPad Software) using one‐way ANOVA. All experiments were performed in at least three replications and *p* < .05 was considered statistically significant.

## 3 RESULTS

### 3.1 Identification of main flavonoid monomers in tartary buckwheat bran extract

**FIGURE 1:**
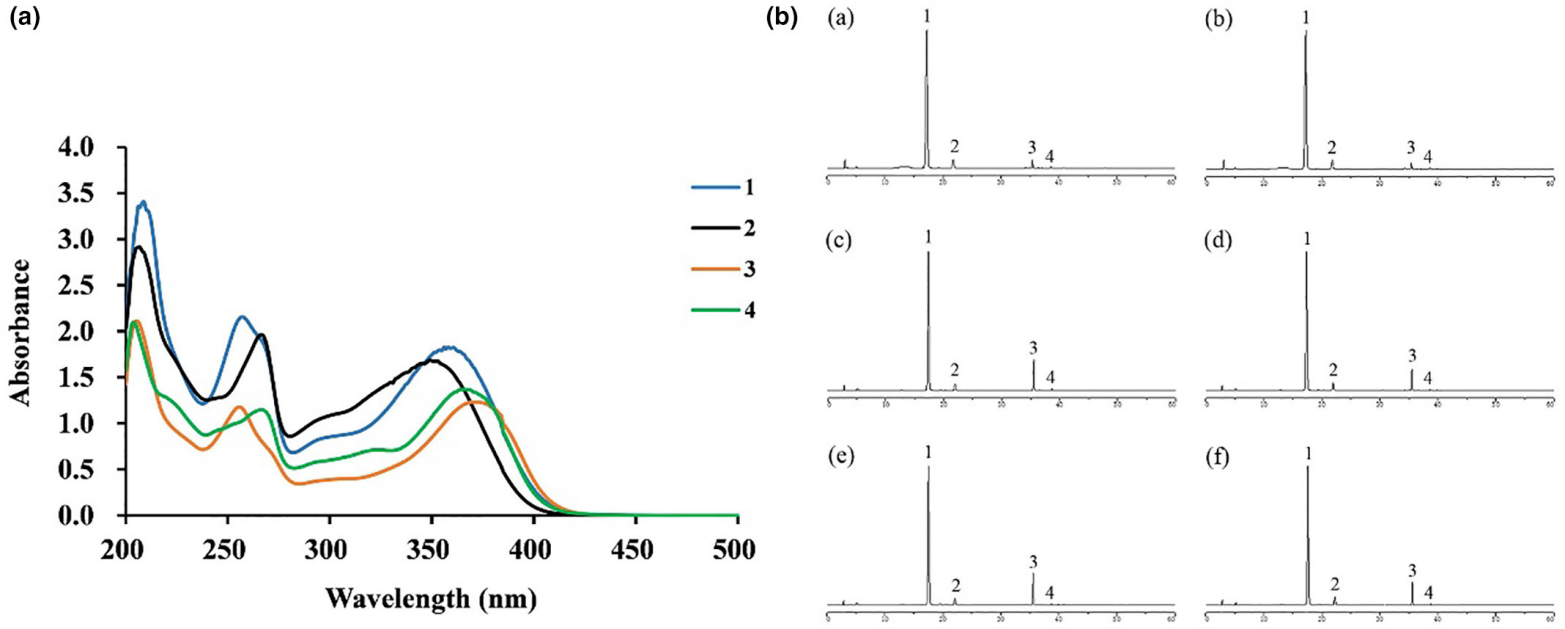
UV–Visible Spectra of the four flavonoid monomer compounds (A); Sample chromatogram map (B) of stage one (a, b), stage two (c, d) and stage three (e, f): (1) rutin; (2) nicotiflorin; (3) quercetin; (4) kaempferol.

As can be seen from Figure 1a, the standard solutions of the four flavonoid monomers had a higher absorption peak around 360 nm overall. At this detection wavelength, we can see that the HPLC maps of the three stages were all very close (Figure 1b). The main flavonoid components of *Fagopyrum Tataricum* bran extract collected were rutin, quercetin, kaempferol, and nicotiflorin, of which the content of rutin was the highest.

### 3.2 Tartary buckwheat flavonoids extract suppressed the growth of *H*. *pylori*

**FIGURE 2:**
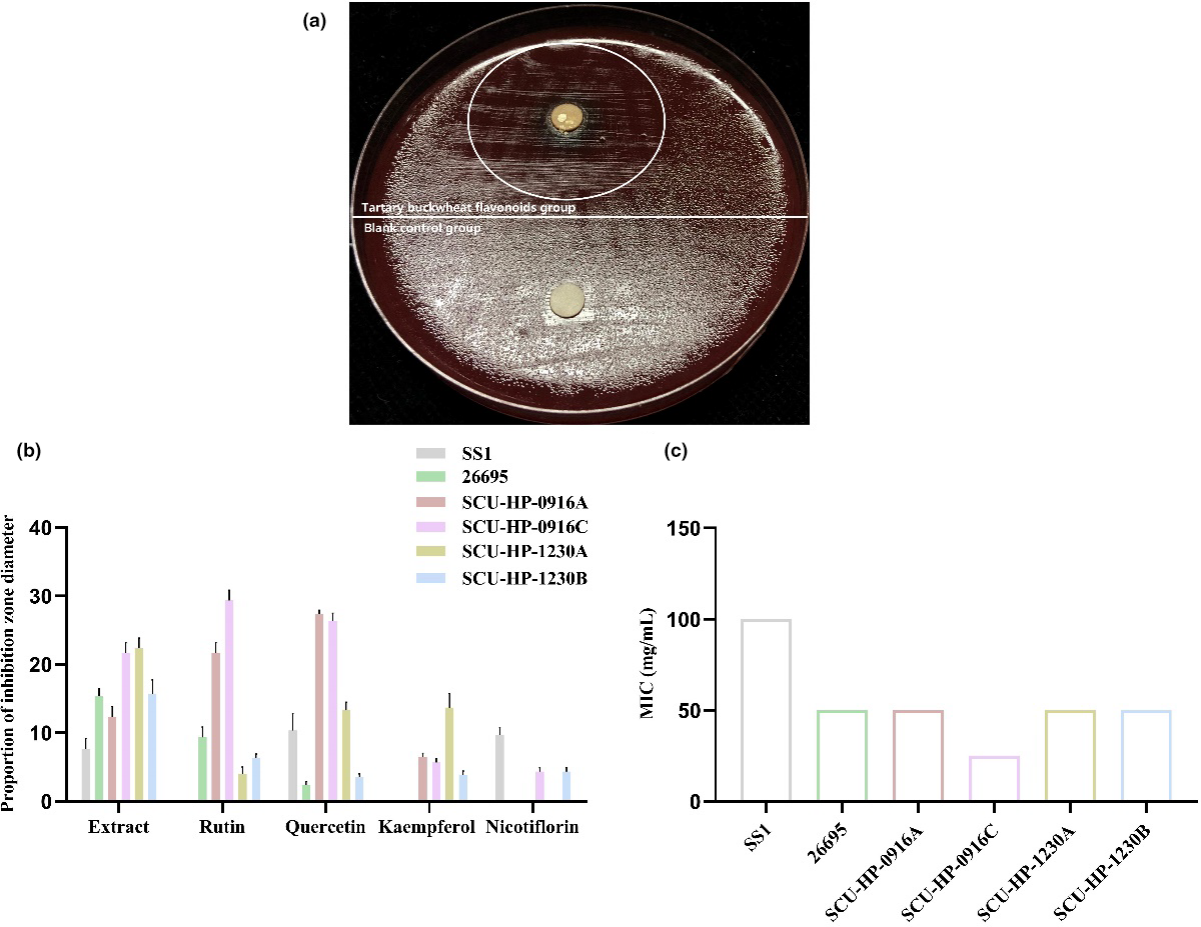
Schematic diagram of the inhibition zone of tartary buckwheat flavonoids extract on *H*. *pylori* (a); proportion of inhibition zone diameter of tartary buckwheat flavonoids extract, rutin, quercetin, kaempferol, and nicotiflorin on *H*. *pylori* (b); MIC of tartary buckwheat flavonoids extract against the six *H*. *pylori* strains (c).

Figure 2 shows that tartary buckwheat flavonoids extract had certain inhibitory activity against the two standard strains (SS1 and 26695) and four clinical strains (SCU‐HP‐0916A, SCU‐HP‐0916C, SCU‐HP‐1230A, and SCU‐HP‐1230B). The schematic diagram of the inhibition zone of tartary buckwheat flavonoids extract on *H*. *pylori* is shown in Figure 2a. Figure 2b exhibits that antibacterial effect on clinical strains was generally better than that on standard strains. In these six tested *H*. *pylori* strains, the strain SS1 showed the weakest inhibitory activity on tartary buckwheat flavonoids extract with the smallest diameter (7.667 ± 0.8819) and the highest MIC (100 mg/mL); the strain SCU‐HP‐0916C showed the strongest inhibitory activity on tartary buckwheat flavonoids extract with the largest diameter (21.67 ± 0.8819) and the lowest MIC (25 mg/mL).

### 3.3 The four flavonoid monomers suppressed the growth of *H*. *pylori*

Figure 2b shows that the four flavonoid monomers (rutin, quercetin, kaempferol, and nicotiflorin) exerted moderate anti‐*H*. *pylori* activity. Same results as tartary buckwheat flavonoids extract, the antibacterial effect of its main flavonoid monomers on clinical strains was generally better than that on standard strains. Moreover, in this experiment, we found that quercetin had a certain antibacterial activity for all tested *H*. *pylori*, which indicated that *H*. *pylori* was highly sensitive to quercetin. A study has reported that quercetin plays an important bacteriostatic role in the extract of Tartary buckwheat bud culture and has the potential to be made as a bacteriostatic agent (Zhong et al., 2022). These results suggested that the presence of the four flavonoid monomers might be the reason why tartary buckwheat flavonoids extract can inhibit the growth of *H*. *pylori*.

### 3.4 UreA and UreB gene expression decreased in *H*. *pylori* after adding tartary buckwheat flavonoids extract

**FIGURE 3:**
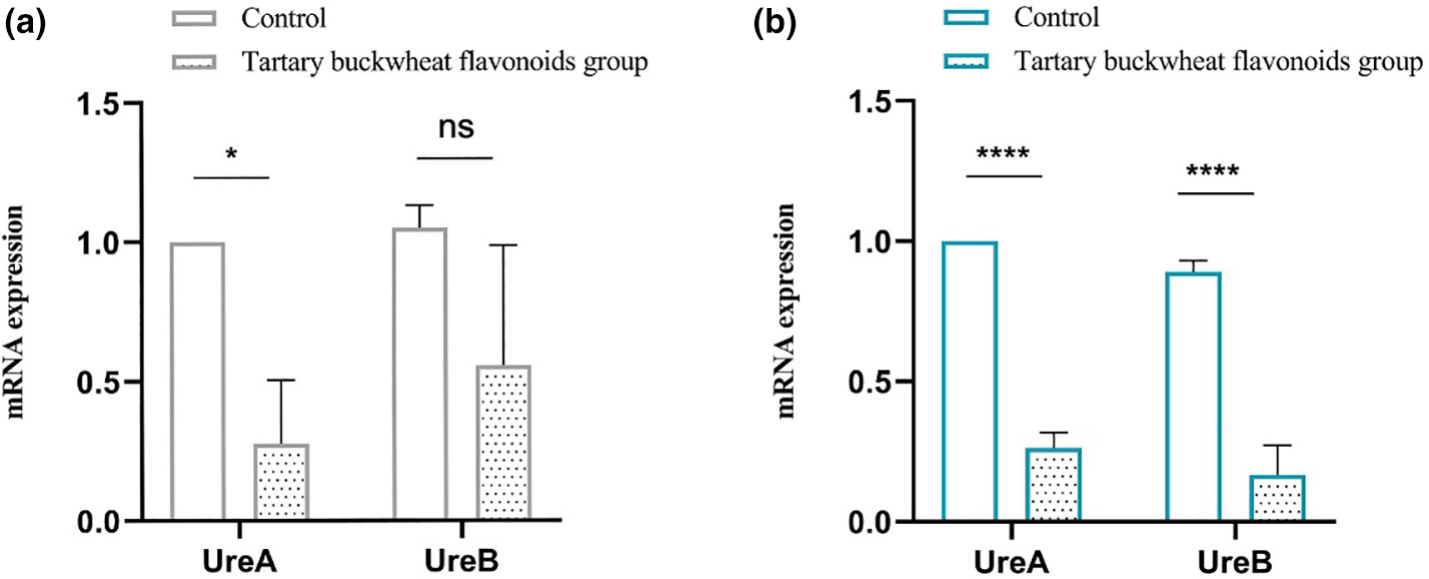
The mRNA expression of UreA and UreB in SS1 (a) and 26695 (b) strains of *H*. *pylori*. The results are the means and SDs (*N* = 3). *p* values: *p < .05; ****p < .0001; and ns *p* > .05.

As shown in Figure 3, compared with the blank control group, the mRNA levels of UreA significantly decreased in SS1 strain (72.3%, *p* < .05) and 26695 strain (73.3%, *p* < .0001) of tartary buckwheat flavonoids group. The mRNA expression of UreB decreased but had no statistically significant difference in SS1 strain (49.3%, *p* = .146), while in 26695 strain, it significantly decreased (72.3%, *p* < .0001). Urease is a cytosolic enzyme which can survive in the extreme acidic environment of the stomach. It plays an important role not only in nitrogen metabolism, but also in acid resistance and virulence (Ghalehnoei et al., 2016). UreA and ureB are two structural subunits of urease and virulence factors of *H*. *pylori*, which can cause strong immune response and can be used as one of the indicators of *H*. *pylori* serological detection (Gómez‐Duarte et al., 1998). After adding tartary buckwheat flavonoids extract, the mRNA expression of UreA and UreB decreased, which suggested that tartary buckwheat flavonoids extract could down‐regulate the virulence factor of *H*. *pylori* and have the potential as urease inhibitor.

### 3.5 Tartary buckwheat flavonoids extract decreased the expression of inflammatory factors in *H*. *pylori*‐infected GES‐1 cells

**FIGURE 4:**
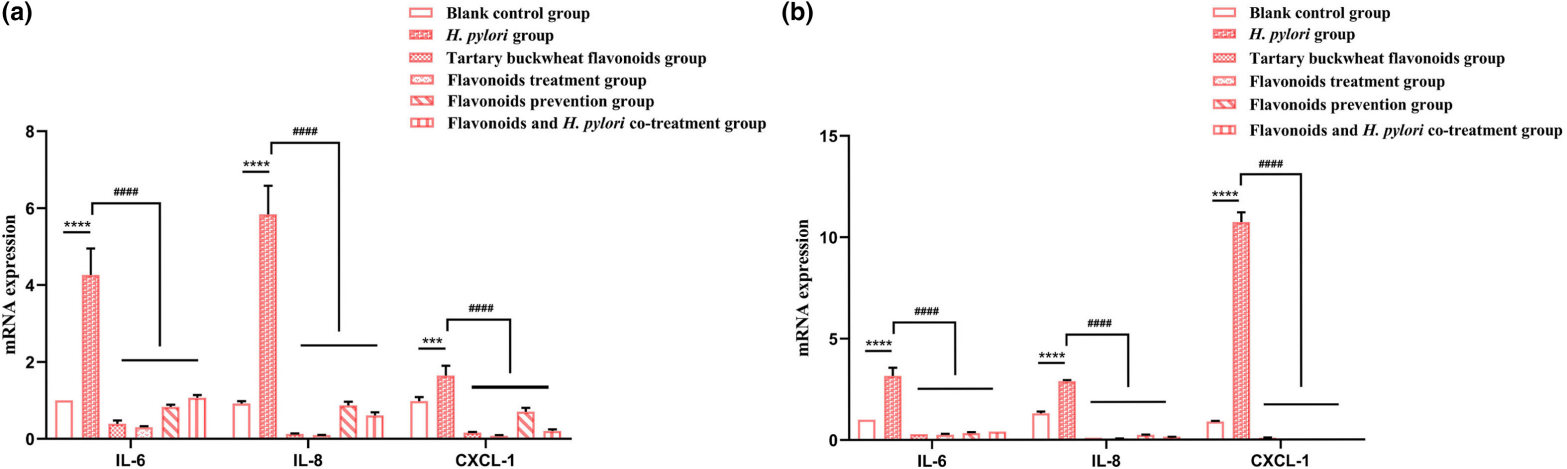
Effects of tartary buckwheat flavonoids extract on inflammatory factors in GES‐1 cells treated with SS1 (a) and 26695 (b) strains of *H*. *pylori*. The results are the means and SDs (*N* = 3). *p* values: ***p < .001; ****p < .0001; and ^####^ *p* < .0001.

Tartary buckwheat flavonoids extract attenuated the inflammatory response caused by *H*. *pylori* infection (Figure 4). Many studies have pointed out that *H*. *pylori* infection can cause the abnormal expression of inflammatory cytokines IL‐6, IL‐8, and CXCL‐1, participate in the production and regulation of intestinal inflammatory diseases, and promote the development of gastric ulcer and even gastric cancer (Deng et al., 2021; Murni et al., 2018). Compared with the blank control group, both the two standard *H*. *pylori* strains (SS1 and 26695) infection significantly increased the mRNA expression of IL‐6 (*p* < .0001), IL‐8 (*p* < .0001, *p* < .05) and CXCL‐1 (*p* < .001, *p* < .0001) in GES‐1 cells, while the tartary buckwheat flavonoids group decreased their mRNA expression (IL‐6: 60.7%, 72.0%; IL‐8: 79%, 92.40%; CXCL‐1: 82.3%, 89.2%). Besides, compared with the *H*. *pylori* group, flavonoids treatment group, flavonoids prevention group, and flavonoids and *H*. *pylori* co‐treatment group all significantly decreased the mRNA expression of IL‐6, IL‐8, and CXCL‐1. The findings from the above experiment revealed that tartary buckwheat flavonoids extract could reduce the mRNA expression of IL‐6, IL‐8, and CXCL‐1 in GES‐1 cells after SS1 and 26695 strains infection. Furthermore, the anti‐inflammatory effect of flavonoids treatment group was better than other groups (IL‐6: 92.9%, 92.0%; IL‐8: 98.8%, 97.2%; CXCL‐1: 95.1%, 99.5%).

### 3.6 Flavonoid monomers decreased the expression of inflammatory factors in *H*. *pylori*‐infected GES‐1 cells

**FIGURE 5:**
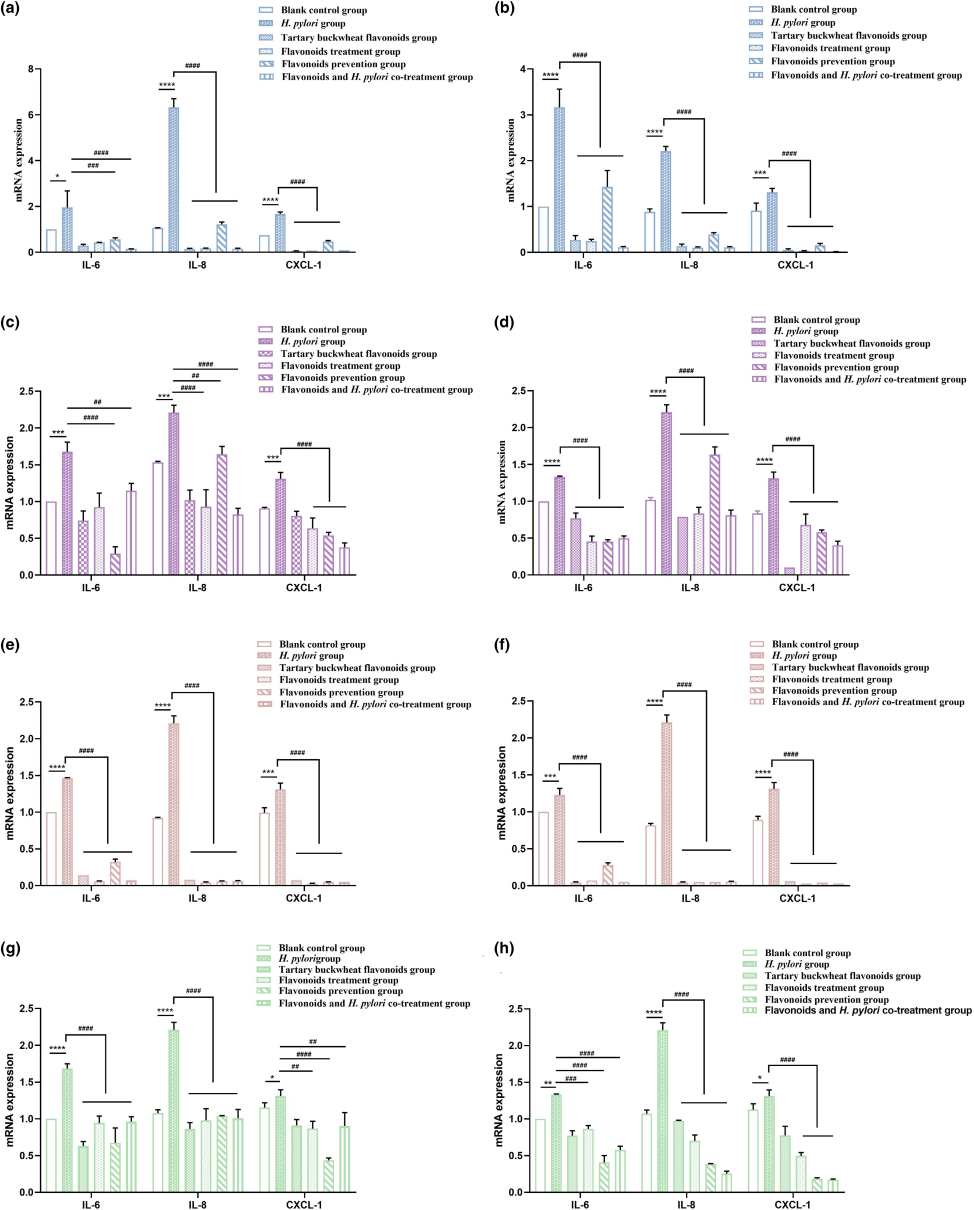
Effects of rutin (a, b), quercetin(c, d), kaempferol (e, f), and nicotiflorin (g, h) on inflammatory factors in GES‐1 cells treated with SS1 (a, c, e, and g) and 26695 (b, d, f, and h) strains of *H*. *pylori*. The results are the means and SDs (*N* = 3). *p* values: *p < .05; **p < .01; ***p < .001; ****p < .0001; ^##^ *p* < .01; ^###^ *p* < .001; and ^####^ *p* < .0001.

As the main flavonoids in tartary buckwheat flavonoids extract, rutin, quercetin, kaempferol, and nicotiflorin reduced the expression levels of IL‐6, IL‐8, and CXCL‐1 secreted by SS1 and 26695 infected GES‐1 cells (Figure 5). Both the two standard *H*. *pylori* strains significantly increased the mRNA expression of IL‐6, IL‐8, and CXCL‐1 in GES‐1 cells compared with the blank control group. Moreover, compared with the *H*. *pylori* group, flavonoids treatment group, flavonoids prevention group, and flavonoids and *H*. *pylori* co‐treatment group had the same trend like tartary buckwheat flavonoids extract. The above results suggested that rutin, quercetin, kaempferol, and nicotiflorin all had a certain inhibitory effect on the cellular inflammation response caused by *H*. *pylori* infection, indicating that these flavonoid monomers may be responsible for the reduction of the inflammatory response caused by *H*. *pylori*.

## 4 DISCUSSION

The rate of *H*. *pylori* infection is increasing, affecting more than half the people in developed countries and 80 percent in underdeveloped countries (Azami et al., 2017). In order to overcome the problem of drug resistance, we urgently need to find alternative drugs to treat *H*. *pylori* infection. Studies have shown that extracts of medicinal plants and herbs have anti‐*H*. *pylori* infections (Ahmed et al., 2021). The application of functional foods may also be an effective approach. Generally, consuming polyphenols triggers reactions in the body, resulting in a degree of anti‐stress and cytoprotective effects (Ruzzolini et al., 2018). As a large class of polyphenols, flavonoids can inhibit energy production, increase cell membrane permeability, and reduce bacterial adhesion, etc. These activities make flavonoids possible as an antimicrobial substance. Tian et al. (2023) found that plant‐based natural flavonoids had strong inhibition on the biosynthesis of *Aspergillus flavus*. Our previous experiments showed that the flavonoids extract of *Fagopyrum Tataricum* had certain inhibitory effects on six common pathogenic bacteria (*Escherichia coli*, *Staphylococcus aureus*, *Salmonella*, *Enterococcus faecalis*, *Shigella*, and *Micrococcus luteum*), and the bacteriostasis abilities of the four flavonoids monomers had a certain structure activity relationship (Wu et al., 2021).

In this study, we quantified the four main monomers of *Fagopyrum Tataricum* bran flavonoids extract by HPLC (Figure 1). Then, through the disk diffusion antimicrobial susceptibility test and the agar dilution method, we found that the flavonoids extract of tartary buckwheat had certain antibacterial activity against different strains of *H*. *pylori*, and the anti‐*H*. *pylori* effect on clinical strains was better than that on standard strains. Besides, the four flavonoid monomers (rutin, quercetin, kaempferol, and nicotiflorin) also had certain antibacterial activity against the standard strains and clinical strains of *H*. *pylori*, respectively. And quercetin had inhibitory effect on all six strains of *H*. *pylori* (Figure 2). Previous studies have shown that flavonoid‐rich plant extracts are effective against drug‐sensitive and resistant strains of *H*. *pylori*, which may be an important alternative or adjunctive treatment (Goswami et al., 2012). It is well known that urease is an important pathogenic factor of *H*. *pylori* (Guo et al., 2014). Actually, the activity of urease helps *H*. *pylori* survive in an acidic environment. It has been shown that inhibition of urease can prevent *H*. *pylori* from adhering to gastric mucosa, which is very important for the treatment of *H*. *pylori*‐related diseases. Besides, inhibiting the activity of urease can inhibit the growth of *H*. *pylori*. Lee et al. (2018) have shown that *Citrus aurantifolia* extract can inhibit the growth and reproduction of *H*. *pylori* by reducing the urease activity. Our study detected the changes of virulence factor mRNA expression after adding tartary buckwheat flavonoids extract into the plates of *H*. *pylori*. The results showed that the supplementation of tartary buckwheat flavonoids extract could reduce the mRNA expression of UreA and UreB genes in the two *H*. *pylori* standard strains, indicating that tartary buckwheat flavonoids extract could down‐regulate the virulence factor of *H*. *pylori* and had the potential as urease inhibitor (Figure 3). The virulence factor of *H*. *pylori* not only has the ability to affect the colonization of organisms and cause disease, but also affects the production of inflammation (Baltas et al., 2016). Persistent inflammatory responses are caused by a variety of proinflammatory factors such as IL‐6, IL‐8, CXCL1. IL‐6 is essential in the occurrence and development of tumors, which can affect cell metastasis and invasion. Through the analysis of serum IL‐6 levels in patients with gastric cancer, it was found that serum IL‐6 could be an independent predictor (Iliopoulos et al., 2009). IL‐8 appears to be associated with histological severity of gastritis and increases tumor cell proliferation, angiogenesis, and cancer cell migration. Eftang et al. (2012) conducted a genome‐wide analysis of gastric epithelial cells infected with *H*. *pylori* for 24 h and found that IL‐8 was the single gene most up‐regulated. CXCL1 regulates host defense against pathogens through multiple pathways and plays an important regulatory role in the transformation, growth, and angiogenesis of gastric cancer cells (Cheng et al., 2011). Jung et al. (2010) found that CXCL1 was overexpressed in patients with gastric cancer, which can be used as a candidate marker for gastric cancer research. Through the different treatments (blank control group, *H*. *pylori* group, tartary buckwheat flavonoids group, flavonoids treatment group, flavonoids prevention group, and flavonoids and *H*. *pylori* co‐treatment group) in this study, we found that the addition of tartary buckwheat flavonoids extract could attenuate the expression of inflammatory factors IL‐6, IL‐8, and CXCL‐1 caused by *H*. *pylori* infection in GES‐1 cells, and the four flavonoid monomers had similar effects after treatments, which indicated that tartary buckwheat flavonoids could prevent and/or treat the inflammatory response caused by *H*. *pylori* infection in gastric epithelial cells (Figures 4 and 5).

## 5 CONCLUSIONS

This study confirmed that tartary buckwheat (*Fagopyrum tataricum* (L.) Gaertn.) flavonoids extract and its main monomers could not only inhibit the growth of *H*. *pylori* in vitro, but also reduce the inflammatory response induced by *H*. *pylori* in GES‐1 cells. Besides, the flavonoids extract could inhibit the expression of urease. The above results indicated that tartary buckwheat flavonoids extract can be used as an effective component in inhibiting the growth of *H*. *pylori* and preventing and treating inflammation caused by *H*. *pylori* infection, which provides a new idea for the development of functional products for health care.

## AUTHOR CONTRIBUTIONS

**Liting Qing:** Writing ‐ Original Draft, Writing ‐ Review & Editing, Formal analysis. **Shu Li:** Methodology, Validation, Formal analysis, Writing ‐ Review & Editing. **Shiying Yan:** Conceptualization, Methodology, Project administration, Supervision. **Chengmeng Wu:** Methodology, Formal analysis, Investigation. **Xin Yan:** Methodology, Formal analysis, Writing ‐ Review & Editing. **Zongyu He:** Writing ‐ Review & Editing. **Qian Chen:** Supervision, Funding acquisition. **Min Huang:** Formal analysis, Investigation. **Caihong Shen:** Writing ‐ Review & Editing. **Songtao Wang:** Resources, Supervision. **Mei Cao:** Supervision, Funding acquisition. **Jian Zhao:** Conceptualization, Writing ‐ Review & Editing, Supervision, Funding acquisition.

## CONFLICT OF INTEREST STATEMENT

The authors have no conflict of interest.

## Data Availability

The data that support the findings of this study are available on request from the corresponding author.
